# The change over time of vital signs with consideration for opioid use in the last 2 weeks of life among cancer patients in a palliative care unit: Continuous measurement of vital signs using a non‐wearable monitor

**DOI:** 10.1002/cam4.4382

**Published:** 2021-11-29

**Authors:** Haruka Tanaka, Sakiko Fukui, Isseki Maeda, Yutaka Hatano, Akari Higuchi, Yoko Higami, Miyae Yamakawa, Momoe Utsumi

**Affiliations:** ^1^ Graduate School of Medicine Osaka University Osaka Japan; ^2^ Department of Integrated Health Sciences, Graduate School of Medicine Nagoya University Aichi Japan; ^3^ Graduate School of Health Science Tokyo Medical and Dental University Tokyo Japan; ^4^ Department of Palliative Care Senri‐Chuo Hospital Osaka Japan; ^5^ Department of Palliative Care Daini Kyoritsu Hospital Hyogo Japan; ^6^ Department of Human Health Sciences, Graduate School of Medicine Kyoto University Kyoto Japan; ^7^ Department of Nursing Osaka Medical and Pharmaceutical University Osaka Japan

**Keywords:** non‐wearable monitor, palliative care, terminal cancer patients, vital signs

## Abstract

**Objectives:**

The aim of this study was to examine the following by using a non‐wearable monitor: (ⅰ) the trajectory of vital signs (VS) in the last 2 weeks of life among cancer patients, and (ⅱ) the difference in change over time of VS between cancer patients with and without opioid use.

**Methods:**

We conducted a longitudinal study involving cancer patients in a palliative care unit (PCU) from April 2018 to October 2019. VS were collected continuously using a non‐wearable monitor, and we calculated the means of respiratory rate (RR) and heart rate (HR) per hour, and counts of apnea per hour as outcome variables. Explanatory variables were time (subtracting time of death from measurement time of VS, divided by 36) and opioid use. Mean difference (MD) of time represented the slope per hour of VS values. First, we analyzed the associations between VS per hour and time using a linear mixed model (LMM) with random intercepts and slope over time. Second, we analyzed the associations between VS and interaction term between time and opioid use.

**Results:**

We analyzed 26 cancer patients. RR (MD: 0.27 beats/min [95% CI: 0.27–0.28]), HR (MD: 1.51 beats/min [95% CI: 1.50–1.52]), and apnea (MD: 0.71 count/hour [95% CI: 0.70–0.72]) significantly increased hourly. In addition, RR was significantly associated with interaction term (MD: −1.59 beats/min [95% CI: −3.11 to −0.07]), which indicates that there is a difference in the slope of RR between opioid users and non‐users.

**Conclusions:**

We have successfully described the trajectory of VS in high‐resolution under conditions of a natural end of life in PCU.

## Graphical abstract

The present study showed that respiratory rate (RR), heart rate (HR), and count of apnea significantly increased over time with adjustment for opioid use in the last 2 weeks of life among cancer patients. In addition, the change over time of RR was different between opioid users and non‐users. This study suggested that RR, HR, and count of apnea were important predictors of impending death for cancer patients.

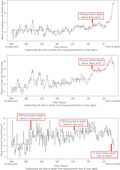

## 1 INTRODUCTION

The leading cause of death in Japan is cancer, where death rates per 100,000 population increased from 235.2 in 2000 to 304.2 in 2019^1^ and continues to increase. In palliative care, maintaining the best possible quality of life (QOL) for cancer patients and their families is imperative. Accurate prediction of impending death or clinical deterioration is useful for patients and their family to plan how to spend end‐of‐life (EOL), which contributes to the maintenance of QOL. Previous studies have examined the prediction of impending death using scales such as the Palliative Performance Scale (PPS) and the Palliative Prognostic Index (PPI)^2^, ^3^ and physical conditions including blood tests and vital signs (VS).^4^, ^5^, ^6^, ^7^ High or increased heart rate (HR) and increased respiratory rate (RR) were associated with impending death.^4^, ^5^, ^6^, ^7^ In addition, our previous study suggested that the cumulative incidence of abnormal RR of 30 or more breaths per minute had a tendency to increase in the last 2 weeks of life among cancer patients in palliative care.^8^ Therefore, HR and respiratory status are important observational parameters in predicting the impending death of cancer patients. However, most previous studies have measured VS discontinuously and have not made known whether HR or RR tend to increase before death. Few, if any, studies have continuously measured VS using non‐wearable monitors and described the detailed trajectory of VS in the last 2 weeks of life among cancer patients.

In palliative care for cancer patients, opioids are often used for pain control. A previous review study showed that the incidence or prevalence of cancer pain was approximately more than 50%, and approximately 64% in advanced, metastatic, or terminal cancer patients.^9^ In a previous study specific to terminal cancer patients in the last 4 weeks of life, 54% of patients had moderate to severe pain (none were pain free), and 97% of patients used opioids.^10^ Opioids sometimes cause respiratory depression and bradycardia. While VS are basic predictors of impending death, they may vary in change over time until death between cancer patients with and without opioid use.

Based on the above, it is important to examine continuous and detailed variation of VS over time until death using a non‐wearable monitor to verify whether VS are valid predictors. In addition, it is important to verify whether there is difference in variation of VS over time between patients with and without opioid use.

The aim of this study was to examine the following by using a non‐wearable monitor: (ⅰ) the trajectory of VS in the last 2 weeks of life among cancer patients, and (ⅱ) the difference in change over time of VS between cancer patients with and without opioid use.

## 2 METHODS

### 2.1 Study design and participants

We conducted a longitudinal study and recruited participants who were hospitalized in a palliative care unit (PCU) in Japan from April 2018 to October 2019. We set up a non‐wearable monitor in the participants’ beds and collected VS continuously for 24 h. We also collected medical and care information from medical records. The inclusion criteria for the participants in this study: (i) men and women who were cancer patients hospitalized in a PCU, (ii) participants whose life expectancy were estimated at a few weeks by palliative care physicians with reference to the PPI,^2^ and (iii) participants who participated in the study until they died and who participated for more than 3 days. The exclusion criteria was participants with missing data from a non‐wearable monitor due to equipment troubles. The present study was approved by the Institutional Review Board of Osaka University (approval number: 1741110). We explained the study protocol and obtained written informed consent from participants. If the participants had difficulty communicating, such as due to cognitive impairment, we explained and obtained written informed consent from a family member who served as a proxy.

### 2.2 Measurements

#### 2.2.1 Outcome variables

The outcome of the present study was vital sign values (RR, HR, and apnea) per hour until death, measured by a non‐wearable monitor (Nemuri SCAN, Paramount Bed Co., Ltd.) placed under the mattress of the participants.^11^, ^12^ The Nemuri SCAN detected the RR, HR, and apnea events by measuring the participants’ body movements every minute. The count of apneas per hour measured by Nemuri SCAN was correlated with these measured by polysomnography used to diagnose apnea.^12^ Using time of death as baseline, we calculated the means of RR and HR per hour, and the count of apneas per hour.

#### 2.2.2 Explanatory variables

The main explanatory of the present study was time and opioid use (with or without). Time (hours) was calculated by subtracting the time of death from the measurement time of VS measurements (i.e., 0, −1, −2, −3…, −336). Regression coefficients of time represented the slope per hour of VS values. The regression coefficient of time is small, so we divided Time by 36 for estimation. Concerning opioid use, we dichotomized participants who used opioids continuously since the start of the measurement or within 3 days of the start and those who did not.

#### 2.2.3 Possible confounders

As positive confounders, we considered the following: (i) age, (ii) sex, and (iii) body mass index (BMI). These were used for the values at the start of the measurement. Age and BMI were used as quantitative variables, and sex as a categorical variable (male: 0, female: 1).

### 2.3 Statistical analysis

We analyzed the data excluding data from 2 h until death because of the possibility that measurements may not have been made correctly due to the many interventions involving medical staff and family. We analyzed VS per hour using a linear mixed model (LMM) including random intercepts and slope.^13^ First, to examine whether VS change over time, we analyzed the associations between VS per hour and time with adjustments for opioid use, age, sex, and BMI (Model 1). In addition, we detected the points at which the mean and variance of VS changed using change‐point analysis.^14^ Second, to examine the difference in change over time of VS between cancer patients with and without opioid use, we analyzed the associations between VS per hour and interaction term between time and opioid use with adjustments for age, sex, and BMI (Model 2).

We obtained standardized coefficients and two‐sided 95% CIs using LMM. For LMM analyses, we used package lme4^15^ in R statistics software version 3.6.1.^16^ For change point analysis, we used package change point^14^ in R statistics software version 3.6.1. LMMs are able to adequately handle missing values of the outcome variable, with a missing‐at‐random (MAR) assumption. For the analysis of missing data, maximum likelihood methods were used as the pattern of the missing data was not MCAR (missing completely at random).

## 3 RESULTS

In this study, 26 adults participated. The characteristics of the participants are summarized in Table 1. The means of the measurement period was 10.23 days, and the means of age was 76.69 years old, ranging from 53 to 95 years of age. For primary cancer sites, “digestive tract” included stomach, large intestine, and esophagus, “gynecological” included uterus and ovary, and “other” included malignant lymphoma and malignant melanoma. We summarized psychotropic medications, including sleeping pills, anti‐anxiety, or anti‐psychotic medications as main medications that affect RR. Four participants continuously used psychotropic medications: two used them in combination with opioid, and two used only psychotropic medications. In 14 opioid non‐users, two participants were of a low consciousness level with little speech ability. Another six participants had pain, but it could be controlled with non‐steroidal anti‐inflammatory drugs (NSAIDs) or acetaminophen. The other six participants had little or no pain.

**TABLE 1:** Characteristics of participants at baseline

Variables	All *n* = 26	Opioid non‐user *n* = 14	Opioid user *n* = 12
Age, mean (SD)	76.88 (10.48)	71.25 (8.52)	81.71 (9.76)
Sex			
Male, *n* (%)	14 (53.85)	7 (50.00)	7 (58.33)
Female, *n* (%)	12 (46.15)	7 (50.00)	5 (41.67)
Body mass index, mean (SD)	18.58 (2.81)	18.24 (2.60)	18.86 (3.05)
Opioid use, *n* (%)	12 (46.15)	—	—
Primary cancer site			
Digestive tract	8 (30.77)	4 (28.57)	4 (33.33)
Pancreas	6 (23.08)	2 (14.29)	4 (33.33)
Hepatobiliary	3 (11.54)	1 (7.14)	2 (16.67)
Breast	3 (11.54)	3 (21.43)	—
Gynecological	2 (7.69)	1 (7.14)	1 (8.33)
Urological	2 (7.69)	1 (7.14)	1 (8.33)
Other	2 (7.69)	2 (14.29)	—

Abbreviation: SD; standard deviation.

**FIGURE 1:**
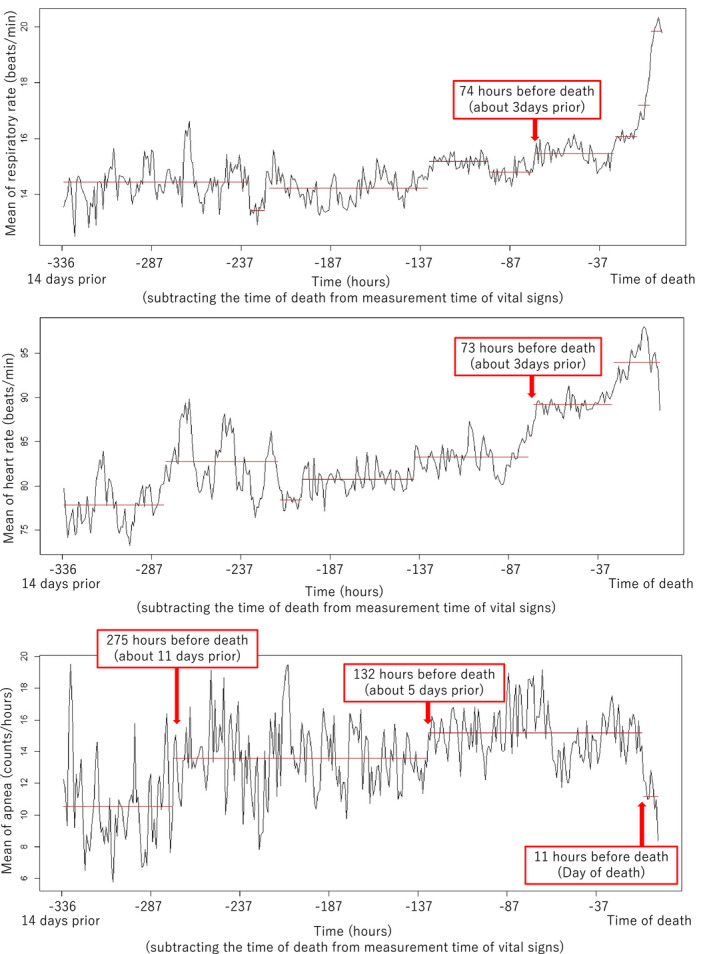
The results of change‐points analysis for detecting the points at which the mean and variance of the vital signs changed

Table 2 shows the results of analysis for the associations between time and VS per hour with adjustments for opioid use and possible confounders using LMM. Respiratory rate (mean difference (MD): 0.27 beats/min [95% CI: 0.27–0.28]), HR (MD: 1.51 beats/min [95% CI: 1.50–1.52]), and apnea (MD: 0.71 counts/hour [95% CI: 0.70–0.72]) significantly increased over time with adjustments for opioid use, age, sex, and BMI in the last 2 weeks of life. In addition, the means of RR and HR of those who used opioids were significantly lower than those who did not at all times (RR [MD: −3.13 beats/min, 95% CI = −5.65 to −0.60], HR [MD: −12.96 beats/min, 95% CI = −24.95 to −0.97]). Figure 1 shows the results of change‐points analysis for detecting the points at which the mean and variance of the VS changed. The break in the red line indicates the change‐point. Respiratory and heart rates showed a marked increase from 3 days before death. Apneas tended to increase from 11 days before death and tended to decrease 11 h before death. Mean and standard deviation of vital signs per day are summarized in the Appendix.

**TABLE 2:** Change over time of vital signs (VS) (Model 1)

	Respiratory rate (beats/min)	Heart rate (beats/min)	Apnea (count/hours)
	Mean difference	Mean difference	Mean difference
	(95% CI)	(95% CI)	(95% CI)
Age (years old)	0.06 (−0.06 to 0.19)	−0.94 (−1.54 to −0.34)	0.14 (−0.23 to 0.50)
Sex (Male = Ref.)	−0.15 (−2.28 to 1.98)	3.46 (−6.65 to 13.58)	1.23 (−4.96. to 7.41)
Body mass index Weight in kg/(height in m)^2^	−0.02 (−0.43 to 0.38)	−1.46 (−3.37 to 0.45)	0.65 (−0.52 to 1.82)
Time^a^ (hours, time of death = Ref.)	0.27 (0.27 to 0.28)	1.51 (1.50 to 1.52)	0.71 (0.70 to 0.72)
Opioid use (without opioids = Ref.)	−3.13 (−5.65 to −0.60)	−12.96 (−24.95 to −0.97)	−2.08 (−9.42 to 5.25)

Note

Age, Sex, BMI, and opioid use represents values collected at the beginning of the measurement.

Mean difference, Mean difference of each VS (Respiratory rate, Heart rate, Number of apneas, Amount of activity) by 1‐unit increase of each explanatory variable; 95% CI, 95% Confidence interval; Ref., Reference.

^a^ Time (hours) was calculated by subtracting the time of death from the measurement time of VS measurements, and the Time was divided by 36.

**FIGURE 2:**
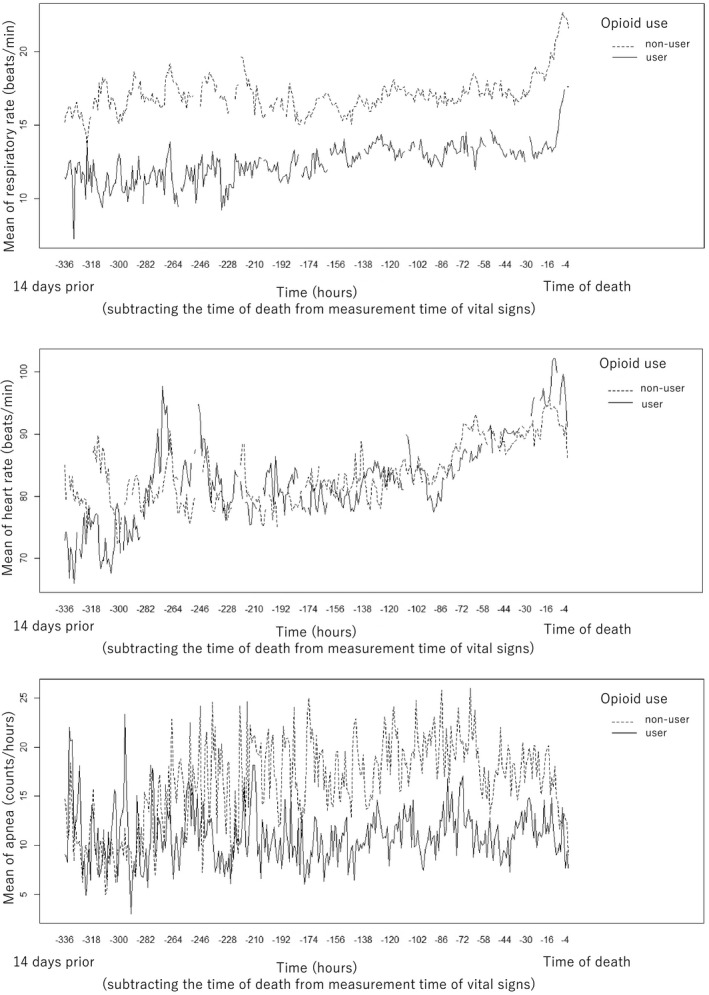
The means of vital signs for each time for opioid users and non‐users, respectively

Table 3 shows the results of analysis for the associations between interaction terms between time and opioid use and VS per hour with adjustments for possible confounders using LMM. RR was significantly associated with interaction term time with opioid use (MD: −1.59, 95% CI = −3.11 to −0.07). HR and apnea were not significantly associated with interaction term. Figure 2 shows the means of VS for each time, for opioid users and non‐users, respectively.

**TABLE 3:** Association between opioid use and change over time of vital signs (VS) (Model 2)

	Respiratory rate (beats/min)	Heart rate (beats/min)	Apnea (count/hours)
	Mean difference	Mean difference	Mean difference
	(95% CI)	(95% CI)	(95% CI)
Age (years old)	0.10 (−0.08 to 0.27)	−0.98 (−1.73 to −0.24)	0.16 (−0.26 to 0.59)
Sex (Male = Ref.)	−0.98 (−3.93 to 1.97)	−0.24 (−12.86 to 12.37)	2.16 (−5.02 to 9.34)
Body mass index Weight in kg/(height in m)^2^	0.12 (−0.44 to 0.67)	−1.49 (−3.88 to 0.89)	1.05 (−0.31 to 2.40)
Time^a^ (time of death = Ref.)	1.73 (0.70 to 2.77)	0.35 (−3.53 to 4.24)	0.61 (−0.48 to 1.70)
Opioid use (without opioids = Ref.)	−4.83 (−8.76 to −0.89)	−9.71 (−26.29 to 6.86)	−4.34 (−14.47 to −5.80)
Interaction term between time and opioid use	−1.59 (−3.11 to −0.07)	2.18 (−3.54 to 7.90)	−0.60 (−2.20 to 1.01)

Note

Age, Sex, BMI, and opioid use represent values collected at the beginning of the measurement.

Mean difference, Mean difference of each VS (Respiratory rate, Heart rate, Number of apneas, Amount of activity) by 1‐unit increase of each explanatory variable; 95% CI, 95% Confidence interval; Ref., Reference.

^a^ Time (hours) was calculated by subtracting the time of death from the measurement time of VS measurements, and Time was divided by 36.

## 4 DISCUSSION

In the present study, we examined the change over time of VS in the last 2 weeks of life among cancer patients, and the difference in change over time of VS between opioid users and non‐users. RR, HR, and apnea significantly increased with adjustments for opioid use, age, sex, and BMI in the last 2 weeks of life. In addition, the means of RR and HR who used opioid were always significantly lower than those who did not. The change over time of RR was different between cancer patients with and without opioid use.

This study showed that HR significantly increased in the last 2 weeks of life among cancer patients with adjustment for opioid use. The result of this study was similar to previous studies: a previous study that developed a short‐term prognostic prediction in advanced cancer patients using multiple logistic regression showed that HR>120/min was associated with death within 7 days^4^; another previous study which investigated predictors for death within 7 days using multiple logistic regression showed that high HR was associated with death within 7 days^5^; a different previous study which examined variation in VS in the last 2 weeks of life in cancer patients showed that HR significantly increased in the last 2 weeks and HR increase>10 was associated with death within 3 days.^7^ Although previous studies did not adjust for opioid use, this study obtained the same result with adjustment for opioid use. Therefore, the result of this study suggests that HR is one of the good predictors of impending death regardless of opioid use.

This study showed that the RR significantly increased in the last 2 weeks of life among cancer patients with adjustment for opioid use. A previous systematic review which evaluated the ability of VS to predict clinical deterioration suggested that RR predicts death.^6^ However, other previous studies have reported that RR was not associated with impending death: a previous study showed that RR was not associated with death within 7 days,^5^ and another previous study showed that RR decrease>5 was not associated with death within 3 days.^7^ These previous studies did not adjust for opioid use, and we, therefore, hypothesized that the discordance in these results was caused by differences in the change over time of RR between opioid users and non‐users. This study demonstrated that the change over time of RR was different between cancer patients with and without opioid use; RR's slope per hour of those who used opioids was more gentle than those who did not. Thus, we should consider the effect of opioid use when using RR as a predictor because the change over time of RR was different between opioid users and non‐users.

Previous studies did not examine the association between apnea and impending death in cancer patients. However, by using a non‐wearable monitor (Nemuri SCAN), we were able to measure the count of apneas per hour in this study. The count of apneas per hour measured by Nemuri SCAN was correlated with these measured by polysomnography used to diagnose apnea.^12^ This study showed that apnea events significantly increased in the last 2 weeks of life among cancer patients with adjustment for opioid use, which suggested that the count of apneas is a good predictor of impending death. However, in clinical settings, not only is the count of apneas important in predicting impending death, but also the duration of apnea events. Development of devices that can measure the duration of apnea is required in the future.

Finally, although change of RR, HR, and count of apnea per hour is small, this study was able to examine the change over time of RR, HR, and count of apnea by using a non‐wearable monitor. In the future, it is necessary to develop a system that can detect changes in RR, HR, and count of apnea in real time; results from this study will serve as basic research for the potential development of such system.

The present study had the following strength. The present study was a longitudinal study continuously measured for 24 h using a non‐wearable monitor, which enabled us to investigate the change over time of VS in the last 2 weeks of life among cancer patients in a PCU. However, this study has several limitations. First, the sample size was small. Second, HR measured by Nemuri SCAN has not yet been validated. Although it is difficult for Nemuri SCAN to accurately measure HR, it is possible to measure changes over time in an individual's HR, and the impact on the results of this study is considerably small. Third, because the number of participants was small, we could not examine medications that affect RR other than opioids. In the future, research is necessary to examine other medications that affect RR. Finally, VS could not be measured when the participants were not in their bed as non‐wearable monitors were used. Most participants were bedridden, but further research is necessary to examine participants who were released from bed during the day such as home care patients.

## 5 CONCLUSIONS

In conclusion, the present study showed that RR, HR, and count of apnea significantly increased over time with adjustment for opioid use in the last 2 weeks of life among cancer patients. In addition, the change over time of RR was different between opioid users and non‐users. This study suggested that RR, HR, and count of apnea were important predictors of impending death for cancer patients. However, we should consider the effects of opioids when using RR as a predictor because the change over time of RR was different between opioid users and non‐users.

## CONFLICT OF INTEREST

The authors have no conflicts of interest to declare.

## ETHICAL APPROVAL STATEMENT

The present study was approved by the Institutional Review Board of Osaka University (Approval number: 1741110). We explained the study protocol and obtained written informed consent from participants. If the participants had difficulty communicating, such as due to cognitive impairment, we explained and obtained written informed consent from a family member who served as a proxy.

## Data Availability

Because of the nature of this research, participants of this study did not agree to the release of their data to the public, therefore supporting data is not available.

## 1

**Table:** TABLE A1 Mean and standard deviation of vital signs per day

Days	Time	Respiratory rate (beats/min) Mean (SD)	Heart rate (beats/min) Mean (SD)	Apnea (count/hours) Mean (SD)
Day of death	Within 24 h of death	17.58 (6.07)	94.33 (20.06)	13.53 (11.71)
2 days ago	25 to 48 h before death	15.38 (4.07)	89.86 (17.65)	14.90 (11.91)
3 days ago	49 to 72 h before death	15.61 (4.41)	89.12 (19.23)	14.81 (11.92)
4 days ago	73 to 96 h before death	14.84 (4.27)	83.31 (16.30)	16.18 (13.59)
5 days ago	97 to 120 h before death	15.14 (3.96)	83.84 (15.42)	14.97 (14.16)
6 days ago	121 to 144 h before death	14.83 (3.75)	82.50 (15.83)	14.06 (12.59)
7 days ago	145 to 168 h before death	14.40 (3.52)	80.96 (15.06)	12.96 (12.72)
8 days ago	169 to 192 h before death	13.92 (3.33)	80.47 (16.85)	14.02 (13.74)
9 days ago	193 to 216 h before death	14.16 (3.29)	79.47 (16.54)	14.30 (13.43)
10 days ago	217 to 240 h before death	14.31 (4.23)	80.90 (17.53)	12.66 (11.56)
11 days ago	241 to 264 h before death	14.33 (4.44)	83.01 (17.06)	14.27 (12.27)
12 days ago	265 to 288 h before death	14.77 (4.14)	82.75 (15.29)	12.30 (9.15)
13 days ago	289 to 312 h before death	14.56 (3.82)	77.30 (12.78)	9.49 (6.66)
14 days ago	313 to 336 h before death	13.96 (3.10)	77.92 (10.23)	11.17 (7.58)

Abbreviation: SD, standard deviation.
